# Comparing the Mosquito-Larvivorous Capacity of the Native Esomus danrica With the Exotic Gambusia affinis and Poecilia reticulata as Biological Vector Control Agents: An Experimental Study

**DOI:** 10.7759/cureus.102344

**Published:** 2026-01-26

**Authors:** Basanta B Das, Dimpymoni Saikia, Diksheeta Chutia, Manoj Talukdar, Dandadhar Sarma

**Affiliations:** 1 Community Medicine, Jorhat Medical College, Jorhat, IND; 2 Zoology, Gauhati University, Guwahati, IND

**Keywords:** biological vector control, esomus danrica, gambusia affinis, larvivorous capacity, larvivorous fish, poecilia reticulata

## Abstract

Background: Larvivorous fishes are important biological vector control agents. *Gambusia affinis* and *Poecilia reticulata* are the most well-known larvivorous fishes used around the world. But introducing exotic fish for biocontrol has adverse long-term impacts. Indigenous fishes with larvivorous potential are more sensible alternatives.

Methodology: Time-bound predation experiments were conducted on three fish species, *G. affinis, P. reticulata,* and *Esomus danrica,* using mosquito larvae as prey. A total of 90 experiments were conducted, 30 on *G. affinis*, 30 on *P. reticulata,* and another 30 experiments on *E. danrica*.

Results: One-way analysis of variance (ANOVA) was conducted to compare the larvivorous capacity of the three fish species. The ANOVA revealed a significant difference in mean scores, *F* (2, 87) = 10.421, *P*-value < 0.001. Post hoc comparison was done using Tukey's Honestly Significant Difference (HSD) test, which revealed a significant difference in larvivorous capacity of *G. affinis *with *P. reticulata* (*P*-value = 0.010) and *E. danrica* (*P*-value = <0.001) individually. However, no significant difference was found in the larvivorous capacity between *P. reticulata *and *E. danrica* (*P*-value = 0.292, effect size (Cohen's *d*) = 0.417 (small)). The mean larval consumption was 11.60 (standard deviation (SD) = 3.635) for *G. affinis*, 9.10 (SD = 2.771) for *P. reticulata*, and 7.83 (SD = 3.291) for *E. danrica*​​​​.​

Conclusions: Our native larvivorous fish (*E. danrica*) showed promising larvivorous capacity. There is a need for further studies that consider additional factors influencing larvivorous capacity, as well as field experiments to determine larvivorous efficacy.

## Introduction

More than 80% of the global population is at risk of vector-borne diseases, with mosquito-borne diseases representing the largest contributor to the human vector-borne disease burden [1]. Vector-borne diseases account for more than 17% of all infectious diseases, resulting in more than one billion cases and over one million deaths each year [2]. Although significant advancements have been made in developing therapeutics and vaccines for mosquito-borne pathogens over the past few decades, efficient vector control strategies remain the primary methods used for controlling and preventing mosquito-borne diseases [3].

Before the use of insecticides, vector control primarily relied on understanding local vector behavioral ecology and implementing tailored environmental control measures. There was also a focus on housing improvements, such as screening of doors and windows, and the use of protective clothing [4]. The first residual pesticide was dichlorodiphenyltrichloroethane (DDT), followed by Dieldrin, Pyrethrin, etc. Initially, they showed promising results, but adverse consequences followed, such as resistance in target populations, hazards to the environment, and public health. Chemical insecticides will still play a significant role in the vector control programme. But the issues they have raised have long sparked interest in alternative vector control measures. Considering this scenario, biological vector control measures are one of the better alternatives. Biological mosquito control measures include the use of pathogens, symbionts, and predators of mosquitoes. Larvivorous fish, known predators of mosquito larvae, are one of the main focuses of biological vector control measures [5]. Nearly complete larval control is possible provided mosquito breeding habitats are well defined, and water conditions are suitable [6]. In the long run, the cost of using larvivorous fish is economical compared with repeated chemical treatment of the same site [7]. The most well-known mosquito-larvivorous fishes are *Gambusia affinis* (commonly known as gambusia or mosquitofish) and *Poecilia reticulata* (commonly known as the guppy) [8]. Both fish species are native to America [9,10]. However, the use of exotic larvivorous fishes as a biocontrol measure has its own threats. *G. affinis* and *P. reticulata* are well known to cause negative ecological impact, such as the extinction of native fishes, amphibians, and several other freshwater communities by outcompeting and preying on these native species. Many of which are natural predators of mosquitoes themselves [11,12]. So, the indigenous larvivorous fishes coexisting in the mosquito larval habitat naturally can serve as a more sensible alternative [13].

The wetland ecosystem of Assam is very rich in fish diversity [14]. There are many indigenous fish with the potential to be used as larvivorous fish [15]. There are certain characteristics of an ideal larvivorous fish. The fish should be small in size so that it can move freely in shallow water among weeds. It should be able to breed freely in confined water bodies; be difficult to catch and capable of escaping natural enemies; be hardy enough to withstand transport and handling stress; survive in shallow waters; be carnivorous, a voracious predator, and preferably a surface feeder; and be able to survive in oxygen-deficient, polluted water. It should preferably be an indigenous fish of the locality [16].​​​​​​​ *Esomus danrica* (locally known as *Dorikona maas* in Assam) has many of these desirable qualities [17]. It is a small fish. The maximum reported length is 6.8 cm, and the weight is 3.4 g. It is found in ditches, paddy fields, wetlands, and rivers. This fish is very active and even able to jump to a considerable height. The mouth is directed obliquely upward, indicating its surface-feeding nature. The species prefers a carnivorous-omnivorous diet. It can be easily identified by maxillary barbels, which are long and extend up to the base of the anal fin. There is a characteristic broad black lateral band extending from behind the eye to the base of the caudal fin [18]. 

In this study, the larvivorous capacity of native fish *E. danrica* was compared with two exotic larvivorous fishes. 

Study objective

To compare the mosquito-larvivorous capacity of the native *E. danrica* with the exotic *G. affinis* and *P. reticulata* under laboratory conditions.

Research question

Is there a significant difference in larvivorous capacity among the native *E. danrica* and the exotic *G. affinis* and *P. reticulata* under laboratory conditions.

Research hypothesis

There is no significant difference in larvivorous capacity among the native *E. danrica, *exotic *G. affinis*, and *P. reticulata* under laboratory conditions.

## Materials and methods

Type of study

It was a laboratory-based experimental study.

Place of study

The study was conducted at the Aquaculture and Biodiversity Centre under the Department of Zoology, Gauhati University, Assam.

Duration of study

The study was conducted from June 18, 2025, to November 30, 2025.

Materials used for the study

Three species of fish, namely *E. danrica*, *G. affinis,* and *P. reticulata,* were used in this study. Mosquito larvae were used as prey. 

The fish were collected from the breeding facility of the Aquaculture and Biodiversity Centre, Department of Zoology, Gauhati University. The fish were kept in rearing tanks before experiments. Each rearing tank was about 100 L in volume. These were made of glass panels fixed with the help of silicone sealant. Three such separate tanks were used for rearing the three fish species. During the rearing period, the fish were fed on a twice-daily schedule, once with commercial fish food and again with mosquito larvae to promote live feeding. The commercial fish food used during the rearing period was of the same brand, type, and size (Piscitek, TATA Pro Fish Feed, manufactured by Rallis India Ltd., Mumbai, India; floating type, pellet size 0.8 mm). The fish were not specific for sex, size, or weight. Within each species, all the fish were full-grown, mature fish. Juvenile fish were excluded from the study. However, no effort was made to match the size among the three fish species.

Mosquito larvae were collected from nearby ditches around Gauhati University, Assam. For this purpose, a dipper fitted with a long handle was used to scoop out the larvae from the water. From the dipper, the larvae were transferred to a plastic tray and later to a plastic container for transportation to the experiment facility. After cleaning the larvae of organic debris, they were kept inside an insect rearing cage in plastic trays till experiment. The insect rearing cage was made of a metal frame and fine mesh. It prevented the accidental escape of adult mosquitoes hatching out of pupae. Mosquito larvae were similarly collected on several occasions till completion of the study. The used mosquito larvae were not specific for species or instar stages.

The experiment tanks were molded acrylic tanks. They had exact markings for measuring 10 L of water. These were the tanks in which the individual experiments were conducted. A total of three such tanks were used throughout the study.

Post-experiment tanks were glass tanks with a volume of approximately 50 L each. They were used to isolate individual fish after the experiments. A total of three such tanks were used, one for each of the three fish species.

Water from all the aquariums in the facility is treated in a single treatment plant and circulated continuously among all the aquariums. Water quality parameters were regularly monitored using a YSI Pro DSS multiparameter probe, maintaining near-optimal conditions (pH 7.6-7.9, temperature 26-28 °C, ammonia 0.058-0.67 mg/L, dissolved oxygen 5.0-5.53 mg/L, total dissolved solids 128.2-235.4 ppm). All the experiments were conducted in daylight conditions.

Handheld scoop nets were used for catching fish from inside the fish tanks. Plastic droppers and fine-tipped watercolor brushes (size 10) were used for handling mosquito larvae. Plastic containers of about 10 L of volume were used for the transportation of mosquito larvae and fish. A digital stopwatch was used for timekeeping during the individual experiments. Glass beakers were used for holding fish or mosquito larvae. Petri dishes were used for holding mosquito larvae after counting.

Sample size

The sample size was purposive. A total of 30 experiments were conducted per species of fish to achieve the minimum sample size for a statistically large sample [19]. A total of 90 experiments were conducted under three groups: 30 experiments on *G. affinis*, 30 experiments on *P. reticulata,* and another 30 experiments on *E. danrica*.

Procedure of the experiments

A total of 90 fish were taken for the 90 experiments: 30 *G. affinis*, 30 *P. reticulata,* and 30 *E. danrica*. During each experiment, 100 mosquito larvae were used, totaling 9,000 larvae across the 90 experiments conducted in the study [20].

Due to the motile nature and small size of mosquito larvae, counting them was a tedious, time-consuming task prone to error. Therefore, the larvae were counted using a silicone mold with 100 cavities, which was placed on a larger tray. A single larva was placed into each cavity with the help of a dropper.

The test fish was put in the 10-L capacity experiment tank filled with acclimatized water from the rearing tank. Further feeding was withheld for 24 hours before the experiment to standardize hunger. A total of 100 mosquito larvae were placed inside this 10-L capacity experiment tank to standardize larval density. The number of mosquito larvae consumed at the end of 30 minutes was counted. This time limit was based on findings from published studies, which reported maximum larval consumption within the first 30 minutes [21]. Time was recorded using a stopwatch.

This same experiment procedure was repeated for all 90 fish, 30 times for *G. affinis*, 30 times for *P. reticulata,* and another 30 times for *E. danrica*. After each experiment, the individual fish was transferred to a post-experiment tank to avoid repeating the experiment on the same fish.

Statistical analysis

Data were analyzed using SPSS version 23 (IBM Corp., Armonk, NY). Levene’s test was conducted to assess the homogeneity of variance among the three groups. The Shapiro-Wilk test was performed to evaluate the normality of the three datasets. Considering fish species as the independent variable and the number of larvae consumed as the dependent variable, a one-way ANOVA was performed to compare the larvivorous capacity among the three fish species. Tukey’s Honestly Significant Difference (HSD) test was conducted as a post hoc analysis to determine significant differences between species. The effect size (Cohen’s *d*) was calculated using the mean larval consumption and the standard deviation of each fish species.

## Results

A total of 90 experiments were conducted under three groups: 30 experiments on *G. affinis*, 30 experiments on *P. reticulata,* and another 30 experiments on *E. danrica*.

Table 1 shows the number of mosquito larvae consumed by the three fish species in 90 individual experiments. Experiments 1-30 correspond to the 30 trials conducted on *G. affinis*, experiments 31-60 correspond to those conducted on *P. reticulata*, and experiments 61-90 correspond to those conducted on *E. danrica*.

**Table 1:** Number of mosquito larvae consumed by the 90 fish across the three species in their 90 individual experiments.

Experiment no.	Larvae consumed (*Gambusia affinis*)	Experiment no.	Larvae consumed (*Poecilia reticulata*)	Experiment no.	Larvae consumed (*Esomus danrica*)
1	18	31	11	61	10
2	12	32	11	62	2
3	4	33	12	63	8
4	7	34	9	64	12
5	8	35	8	65	3
6	12	36	6	66	4
7	11	37	5	67	10
8	12	38	9	68	11
9	10	39	7	69	9
10	9	40	12	70	11
11	8	41	8	71	3
12	13	42	6	72	7
13	11	43	12	73	8
14	15	44	7	74	3
15	18	45	11	75	11
16	12	46	12	76	12
17	6	47	8	77	14
18	13	48	11	78	8
19	11	49	10	79	13
20	14	50	15	80	9
21	11	51	6	81	5
22	18	52	12	82	6
23	18	53	12	83	10
24	12	54	6	84	6
25	6	55	4	85	8
26	11	56	5	86	9
27	12	57	12	87	4
28	15	58	8	88	5
29	9	59	8	89	9
30	12	60	10	90	5

Considering the total number of larvae consumed in 30 experiments, *G. affinis* consumed 348 larvae, followed by *P. reticulata* with 273 larvae, and *E. danrica* with 235 larvae. The mean larval consumption was 11.60 (SD = 3.635) for *G. affinis*, 9.10 (SD = 2.771) for *P. reticulata*, and 7.83 (SD = 3.291) for *E. danrica*, as shown in Table 2.

**Table 2:** Descriptive statistics of the number of mosquito larvae consumed by each fish species in 30 species-wise experiments.

Fish species	Number of experiments in each group	Sum of larvae eaten in 90 experiments	Mean larvae consumed	Standard deviation	Minimum number of larvae consumed	Maximum number of larvae consumed
Gambusia affinis	30	348	11.60	3.635	4	18
Poecilia reticulata	30	273	9.10	2.771	4	15
Esomus danrica	30	235	7.83	3.291	2	14
Total	90	856	9.51	3.580	2	18

The Levene’s test result (Levene’s statistic = 0.329, *P* = 0.721) for the larval consumption data indicates homogeneity of variance among the three groups. The Shapiro-Wilk test results were: *G. affinis*, *W* = 0.951, *P* = 0.178; *P. reticulata*, *W* = 0.945, *P* = 0.121; and *E. danrica*, *W* = 0.964, *P* = 0.385, indicating that the data for all three groups were normally distributed. All three species-wise groups of experiments (30 experiments for each species of fish) were independent of one another. A one-way ANOVA test was performed to compare the mean larval consumption within a time limit of 30 minutes by the three fish species. The ANOVA test revealed a significant difference in mean scores, *F* (2, 87) = 10.421, *P*-value < 0.001, as shown in Table 3.

**Table 3:** Summary of ANOVA test findings. ANOVA, analysis of variance

Source	Sum of squares	Degree of freedom	Mean sum of squares	*F*-value	*P*-value
Between groups	220.422	2	110.211	10.421	<0.001
Within groups	920.067	87	10.575		
Total	1140.489	89			

Post hoc comparisons were conducted using Tukey’s HSD test, which revealed a significant difference in the larvivorous capacity of G. affinis compared with P. reticulata (P = 0.010) and E. danrica (P = <0.001). However, no significant difference was observed between P. reticulata and E. danrica (P = 0.292) (Table 4).

**Table 4:** Summary of Tukey's HSD test findings. *The mean difference is significant at the 0.05 level. HSD, Honestly Significant Difference

Species of fish (I)	Species of Fish (J)	Mean Difference (I-J)	Standard Error	Significance (p-value)	95% Confidence interval	
					Lower Bound	Upper Bound
Gambusia affinis	Poecilia reticulata	2.500^*^	0.840	0.010	0.50	4.50
Gambusia affinis	Esomus danrica	3.767^*^	0.840	<0.001	1.76	5.77
Poecilia reticulata	Esomus danrica	1.267	0.840	0.292	-0.74	3.27

The effect size (Cohen’s *d*) was calculated to quantify the magnitude of differences in larval consumption between the species. Cohen’s *d* between *G. affinis* and *P. reticulata* was 0.774 (medium). Similarly, the effect size between *G. affinis* and *E. danrica* was 1.087 (large), and between *P. reticulata* and *E. danrica* was 0.417 (small). The classifications of small, medium, and large effect sizes are based on Jacob Cohen’s guidelines [22].

## Discussion

This study was conducted to compare the mosquito-larvivorous capacity of native *E. danrica* with that of exotic *G. affinis* and *P. reticulata*. Time-bound predation experiments were conducted using mosquito larvae as prey.

Very few studies are available that compare the larvivorous capacity of these three species together. For the discussion, we included only those studies that investigated at least two of the three fish species.

The total sample size per group was purposively set at 30, fulfilling the requirement for a minimum sample size sufficient for robust statistical analysis [19].

A one-way ANOVA was conducted, with fish species as the independent variable and the number of larvae consumed as the dependent variable. A significant difference in larvivorous capacity was observed among the three fish species, *F*(2, 87) = 10.421, *P* < 0.001.

Further analysis using Tukey’s HSD test revealed a significant difference in larvivorous capacity between *G. affinis* and *P. reticulata*, with *G. affinis* consuming more mosquito larvae. This finding is consistent with the study conducted by Sangeetha et al. However, unlike our study, their research used fourth-instar Culex mosquito larvae as prey and assessed larvivorous capacity over 24 hours [23].

We also found a significant difference in the larvivorous capacity between *G. affinis* and *E.* *danrica*, where *G. affinis* consumed more mosquito larvae. This finding was also consistent with the study conducted by Bano and Serajuddin [20]. In their study, as in ours, 100 mosquito larvae were provided as prey, but for a duration of 2 hours.

In our study, we did not find any significant difference in larvivorous capacity between *P. reticulata* and *E. danrica,* which was dissimilar to the findings of the study by Aditya et al. [24]. They found a significant difference in larvivorous capacity between the two fish, with *E. danrica* consuming more mosquito larvae. Further, in the same study, a variety of food items, including commercial fish food and live feed such as mosquito larvae, tubifex worms, and chironomid larvae, were introduced simultaneously to assess preference for mosquito larvae in the presence of alternative food. In that study, *P. reticulata *preferred commercial fish food, whereas *E. danrica* preferred live feed such as mosquito larvae, tubifex worms, and chironomid larvae. Both our study and the study conducted by Aditya et al. found *E. danrica* to be suitable as a larvivorous fish [24].

The larvivorous capacity of *E. danrica* was significantly less than that of *G. affinis*. But it showed promising larvivorous capacity compared to *P. reticulata*. *E. danrica*, a native fish, has the added advantage of being free from long-term ecological adverse effects, unlike its exotic counterparts [11-13]. Thus, this fish can be considered for the biocontrol of mosquito vectors, at least within its native area of distribution. However, before implementation, further laboratory experiments on *E. danrica* are needed, considering additional factors that influence larvivorous capacity, followed by field experiments in natural mosquito habitats.

Limitations of the study

The sample size was purposively set at 30 per fish species to achieve the minimum required sample size for adequate statistical analysis [19].

Fish characteristics, such as size, sex, age, weight, etc., could not be standardized due to operational feasibility.

Mosquito larvae instar stages and species could not be standardized due to operational feasibility.

All experiments were conducted under daylight conditions; however, the light intensity was not quantified. The presence of alternative food sources was not considered [25].

## Conclusions

Our study found a significantly higher larvivorous capacity in *G. affinis* compared to *P. reticulata* and *E. danrica*. However, no significant difference was observed between *P. reticulata* and *Esomus danrica*. In this study, our native larvivorous fish *E. danrica* could not outcompete *G. affinis*; however, its larvivorous capacity was comparable to that of *P. reticulata*. Being a native species, E. danrica is free from the adverse ecological outcomes associated with exotic counterparts. The fish *E. danrica* can be considered for the biocontrol of mosquito vectors, at least within its native area of distribution. However, our study was laboratory-based, in which larvivorous capacity was assessed under controlled conditions. Laboratory-based assessments of larvivorous capacity may not reflect true larvivorous efficacy under real-life field conditions. There is a need for further laboratory studies that consider additional factors influencing larvivorous capacity, followed by field experiments to determine larvivorous efficacy in real-world mosquito habitats.
